# Bone marrow adipose tissue does not express UCP1 during development or adrenergic-induced remodeling

**DOI:** 10.1038/s41598-019-54036-x

**Published:** 2019-11-22

**Authors:** Clarissa S. Craft, Hero Robles, Madelyn R. Lorenz, Eric D. Hilker, Kristann L. Magee, Thomas L. Andersen, William P. Cawthorn, Ormond A. MacDougald, Charles A. Harris, Erica L. Scheller

**Affiliations:** 1 Division of Bone and Mineral Diseases, Department of Internal Medicine, Washington University School of Medicine, Saint Louis, MO USA; 2 Department of Cell Biology & Physiology, Washington University School of Medicine, Saint Louis, MO USA; 3 Division of Endocrinology, Metabolism, and Lipid Research, Department of Medicine, Washington University School of Medicine, St. Louis, MO USA; 4 Department of Medicine, Veterans Affairs St. Louis Healthcare System, John Cochran Division, St. Louis, MO USA; 5 Department of Pathology, Odense University Hospital – Department of Clinical Research & Department Molecular Medicine, University of Southern Denmark, Odense, Denmark; 6 Department of Forensic Medicine, Aarhus University, Aarhus, Denmark; 7 University/BHF Centre for Cardiovascular Science, The Queen’s Medical Research Institute, Edinburgh Bioquarter, University of Edinburgh, Edinburgh, UK; 8 Department of Molecular & Integrative Physiology, University of Michigan, Ann Arbor, MI USA

**Keywords:** Mitochondrial proteins, Genetic models, Cell biology, Cell signalling, Mechanisms of disease

## Abstract

Adipocytes within the skeleton are collectively termed bone marrow adipose tissue (BMAT). BMAT contributes to peripheral and local metabolism, however, its capacity for cell-autonomous expression of uncoupling protein 1 (UCP1), a biomarker of beige and brown adipogenesis, remains unclear. To overcome this, *Ucp1*-Cre was used to drive diphtheria toxin expression in cells expressing UCP1 (*Ucp1*^Cre+/DTA+^). Despite loss of brown adipose tissue, BMAT volume was not reduced in *Ucp1*^Cre+/DTA+^ mice. Comparably, in mTmG reporter mice (*Ucp1*^Cre+/mTmG+^), *Ucp1*-Cre expression was absent from BMAT in young (3-weeks) and mature (16-weeks) male and female mice. Further, β3-agonist stimulation failed to induce *Ucp1*-Cre expression in BMAT. This demonstrates that BMAT adipocytes are not UCP1-expressing beige/brown adipocytes. Thus, to identify novel and emerging roles for BMAT adipocytes in skeletal and whole-body homeostasis, we performed gene enrichment analysis of microarray data from adipose tissues of adult rabbits. Pathway analysis revealed genetic evidence for differences in BMAT including insulin resistance, decreased fatty acid metabolism, and enhanced contributions to local processes including bone mineral density through candidate genes such as osteopontin. In sum, this supports a paradigm by which BMAT adipocytes are a unique subpopulation that is specialized to support cells within the skeletal and hematopoietic niche.

## Introduction

Bone marrow adipose tissue (BMAT) is the collection of adipocytes found within medullary cavities of the skeleton. In humans, rapid expansion of BMAT begins postnatally with BMAT occupying up to 70% of medullary space by age 23, giving bone marrow a yellow appearance^1^. Cross-sectional clinical studies have associated increased BMAT with decreased bone density and increased fracture incidence in elderly populations (reviewed in^2^). However, it is important to highlight that peak bone mass in humans is attained coincident with rapid BMAT expansion indicating that BMAT is not uniformly harmful to bone^3^. Similar to humans, physiological BMAT expansion in rodents occurs coincident with bone accrual, and comparison of BMAT across mouse strains reveals bone mass and BMAT volume can be positively correlated in states of health. Specifically, C3H/HeJ mice have both higher bone mass and BMAT volume compared to C57BL/6J mice^4^. By contrast, animal studies during pathologic conditions have demonstrated that BMAT volume is often, but not always, associated with impaired bone mass or quality (reviewed in^5^). Beyond bone, BMAT has the capacity to function as an endocrine organ and site of energy storage and release^6,7^. Thus, additional characterization of BMAT development, regulation and function during both healthy and pathologic conditions is necessary to facilitate our understanding of BMAT’s relationship both to bone and to peripheral metabolism.

Extraskeletal adipocytes are grouped into functional classifications which range from brown or beige, to white and pink (reviewed in^8^). Even within a subclass, for example white adipose tissues (WAT), metabolic responsiveness can vary widely between depots reflecting diverse functions of adipocytes in both structural support and lipid partitioning (reviewed in^9^). In post-pubescent mice, BMAT adipocytes have been classified into two categories, regulated BMAT (rBMAT) and constitutive BMAT (cBMAT) (reviewed in^10^). Regulated BMAT adipocytes reside in diverse, multicellular niches which can include pericytes, hematopoietic cells and osteogenic cells^11^. These cells are capable of lipid droplet remodeling in response to β3-agonist stimulation, however, BMAT lipid droplet remodeling is modest and morphologically distinct from ‘beige-like’ remodeling traditionally observed in white adipose tissues^7^. Constitutive BMAT adipocytes reside in homologous cBMAT niches and are relatively resistant to adrenergic stimuli^7^.

Though bone marrow adipocytes generally resemble white adipocytes morphologically, whether BMAT adipocytes are ‘beige-like’ remains unclear. Fundamental characteristics of ‘beige’ adipocytes include their ability to remodel from a unilocular to multilocular morphology and their capacity for induced expression of uncoupling protein-1 (UCP1), a biomarker of thermogenesis^12^. UCP1 is a key brown/beige adipocyte protein that resides on the inner mitochondrial membrane where its presence allows energy released from fatty acids to be converted into heat^13^. In support of BMAT beiging, adipocytes in the proximal regions of the mouse skeleton (rBMAT) have been reported to express markers of brown fat, including *Ucp1*, at the level of RNA transcript^14–17^. *In vivo*, there is one report showing a multilocular appearing cell in the lumbar vertebrae of a developing mouse with immunohistochemical evidence that this cell expressed UCP1 protein^18^. Also *in vivo*, treatment of female C3H/HeJ mice with ß3-adrenergic agonist CL316,243 has been shown to induce modest lipid droplet remodeling, resulting in a multilocular morphology, within a subpopulation of rBMAT adipocytes^7^. Finally, lineage tracing has provided anecdotal evidence that a subset of BMAT adipocytes have beiging potential. Specifically, BMAT adipocytes have been shown to express *PdgfRα*, a mediator of adipocyte progenitor commitment to a beige adipocyte^19^, following irradiation or rosiglitazone treatment (discussed in^20^).

Arguing against BMAT beiging includes a limitation in the above mentioned *Ucp1* transcript studies. *Ucp1* transcript was measured from RNA acquired from whole bone preparations which are highly heterogeneous and susceptible to contamination from extraskeletal adipocyte-containing soft tissues. In addition, the amount of *Ucp1* transcript detected was significantly less than found in BAT (>10,000 fold)^14,16^. Second, the majority of brown adipocyte progenitors and a minority of beige progenitors express myogenic factor-5 (*Myf5*)^21,22^, however, lineage tracing has shown that BMAT progenitors do not express *Myf5* (discussed in^20^). Finally, there is also preliminary *in vivo* evidence that UCP1 protein expression is absent in BMAT adipocytes. Specifically, a methionine-restricted diet was used to induce beiging of white adipocytes. This diet successfully resulted in increased UCP1 protein expression in inguinal white adipose tissue (a predominant site of beige adipocytes in rodents) but, unlike the findings of Nishino *et al*.^18^, failed to induce UCP1 protein expression by IHC in BMAT adipocytes^20,23^. Thus, it is clear that significant controversy exists in the literature regarding this point.

To overcome this, we sought to track UCP1 expression in BMAT at the single cell level using multiple rodent models of development and adrenergic-stimulated remodeling. Our laboratory has previously observed BMAT adipocytes in adult mice containing multiple lipid droplets using 3D electron microscopy^11^, as well as modest lipid droplet remodeling in response to β3-agonist stimulation^7^; both are characteristics of beige adipocytes^7,11^ and of white adipocytes that are developing or undergoing stimulated lipolysis (reviewed in^24^). Until now, we had not definitively addressed whether BMAT adipocytes are truly beige-like adipocytes as defined by their capacity to express UCP1. Clarification of whether some BMAT adipocytes express UCP1 has important implications for our understanding of the role of BMAT in skeletal processes including hematopoiesis and bone turnover. Here, immunohistochemical studies were coupled with genetic ablation and lineage tracing of UCP1 expressing cells to determine whether any BMAT adipocyte population expressing UCP1 exists during development, in adulthood, or after systemic β3-adrenergic stimulation. In addition, to define novel pathways that separate BMAT and WAT, we performed serial gene enrichment and pathway analysis of microarrays from both white and bone marrow adipose tissues.

## Results

### Bone marrow adipocytes exist within a sinusoidal niche and are predominately unilocular

**Figure 1:**
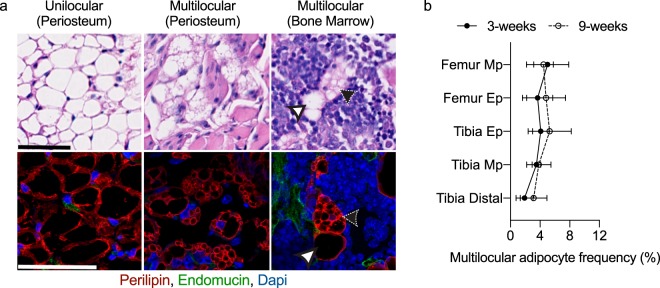
Bone marrow adipocytes exist within a sinusoidal niche and are predominately unilocular. (**a**) Representative images of unilocular and multilocular adipocytes located outside the bone, near the periosteal bone surface (periosteum) or within the bone marrow of 3-week-old C57BL/6J mouse femurs and tibias Hematoxylin and eosin images (top) were acquired at 40X, scale bar is 50 µm. Immunofluorescent confocal images (bottom) were acquired at 60X, scale bar is 50 µm. Adipocytes were visualized with α-perilipin antibody (red), sinusoids were visualized with α-endomucin antibody (green), and nuclei were visualized with DAPI (blue). Unilocular and multilocular adipocytes are indicated by white and black arrowheads, respectively. (**b**) Regional analysis of multilocular adipocyte frequency (relative to total adipocyte number), in 3-week-old and 9-week-old C57BL/6J mouse bones. Metaphysis (Mp), epiphysis (Ep). Mean ± SD. Sample sizes: 3-week n = 4–6, 9-week n = 6–7.

The lipid droplet morphology and localization of BMAT adipocytes in the developing limb was examined in male and female C57BL/6J mice. Adipocytes and vascular sinusoids were visualized using perilipin and endomucin immunohistochemistry, respectively. At 3-weeks of age, bone marrow adipocytes were nearly absent in the metaphyseal regions of the distal femur and the proximal metaphysis of the tibia. By contrast, prominent marrow adipocyte populations were noted in the distal tibia and in both the proximal tibia and distal femoral epiphyses. At 3-weeks of age, both unilocular and multilocular adipocytes were found on the exterior of the bone, near the periosteal surface (Fig. 1a). Multilocular adipocytes near the periosteum were largely observed in the proximal tibia-fibula junction and near the femoral neck. Within the bone marrow, it was observed that endomucin positive endothelium forms a peri-sinusoidal niche for most, if not all, of the bone marrow adipocytes. The predominant morphology of the BMAT adipocytes was a single, large perilipin + lipid droplet with an eccentric nucleus (Fig. 1a). However, a subset of the BMAT adipocytes in both femur and tibia had multiple small perilipin positive lipid droplets positioned around a central nucleus (Fig. 1a). The frequency of multilocular bone marrow adipocytes in 3-week-old mice (~5%) was found to be comparable across all regions of the femur and tibia (Fig. 1b). This finding remained constant even in postpubescent animals at 9-weeks of age (Fig. 1b).

### Immunostaining for UCP1 in BMAT is inconclusive

**Figure 2:**
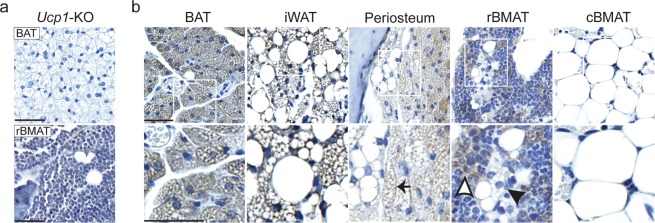
Immunostaining for UCP1 in BMAT is inconclusive. (**a**) IHC of brown adipose tissue (BAT, top) and rBMAT (bottom) from *Ucp1*-knockout mouse (*Ucp1*-KO, C57BL/6J) using α-UCP1 antibody. Images were acquired at 40X, scale bar is 50 µm. (**b**) Images of brown adipose tissue (BAT), inguinal white adipose tissue (iWAT), adipocytes external to the bone, near the periosteal surface (periosteum), cBMAT (tail), and rBMAT from C3H/HeJ mice treated with 5 μg CL316,243 per mouse per day for 3-days. Images were acquired at 40x (top) or 80x (bottom), scale bar is 50 µm. White boxes in top images show inset for below images. White arrowhead indicates non-specific staining. Black arrowhead highlights a multilocular BMAT adipocyte with indeterminate UCP1 expression. Black arrow highlights multilocular BAT-like cells near the periosteal surface. These cells are positive for UCP1 immunostaining.

UCP1 protein expression was examined in BMAT and peripheral adipose tissues using immunohistochemistry. When calibrated using tissue from *Ucp1*-knockout mice (Fig. 2a), immunostaining for UCP1 was positive in peripheral brown adipocytes of interscapular brown fat (BAT) and beige adipocytes within inguinal white adipose tissue (iWAT) (Fig. 2b). Cells with a morphology similar to beige adipocytes (multiple small lipid droplets associated with large lipid droplet^7^) which stained positive for UCP1 were also found near the periosteal surface, most commonly seen in the femoral neck and proximal tibia-fibula junction. This finding indicates that whole bone RNA preparations are susceptible to contamination by UCP1-positive adipocytes near the periosteal surface. Within the bone, UCP1 staining appeared negative in tail BMAT regions (cBMAT). However, non-specific staining in BMAT regions rich in hematopoietic cells (rBMAT) made definitive conclusions on UCP1 expression in rBMAT challenging (Fig. 2b). Thus, we opted to move forward with the two genetic mouse models described below.

### Genetic ablation of UCP1-expressing cells does not cause loss of BMAT at 4-weeks of age

**Figure 3:**
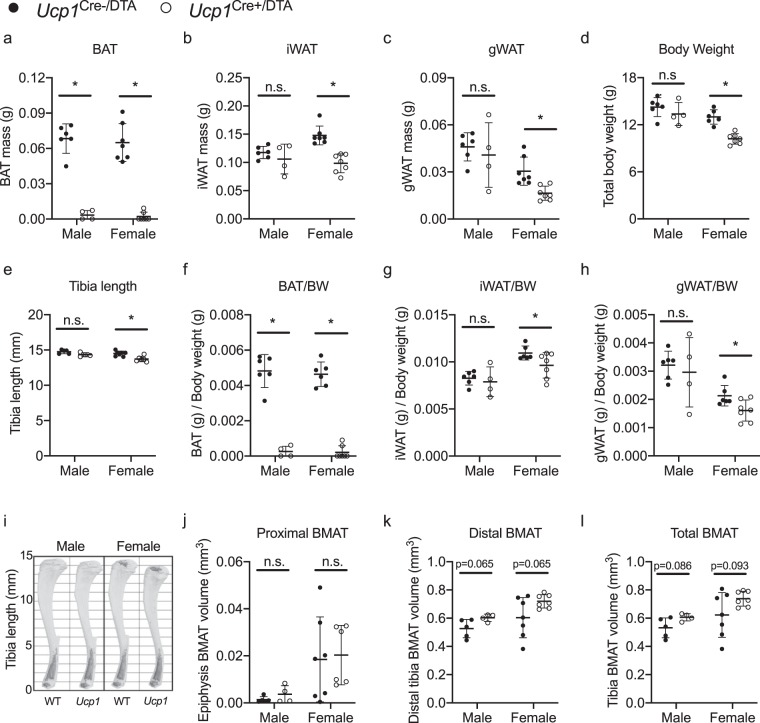
Genetic ablation of *Ucp1*-Cre expressing cells results in loss of BAT but not BMAT. (**a**–**c**) Tissue weights for *Ucp1*^Cre−/DTA^ (WT) and *Ucp1*^Cre+/DTA^ brown adipose tissue (BAT), inguinal white adipose tissue (iWAT), and gonadal white adipose tissue (gWAT). (**d**) *Ucp1*^Cre−/DTA^ and *Ucp1*^Cre+/DTA^ mouse total body weight (BW). (**e**) *Ucp1*^Cre−/DTA^ and UCP1^Cre+/DTA^ tibia length measured by digital caliper. (**f**–**h**) *Ucp1*^Cre−/DTA^ and *Ucp1*^Cre+/DTA^ BAT, iWAT and gWAT tissue weights normalized to total body weight (BW). (**i**) Representative 3-D images of osmium stained bones to visualize *Ucp1*^Cre−/DTA^ (WT) and *Ucp1*^Cre+/DTA^ (*Ucp1*) bone marrow adipose tissue (BMAT). Light grey is outline of bone. Dark grey is osmium-stained BMAT. Analysis and 3-D rendering of the μCT data sets were performed using Scanco software. (**j**–**k**) Regional analysis of osmium content in decalcified *Ucp1*^Cre−/DTA^ and *Ucp1*^Cre+/DTA^ tibias. (**j**) Proximal BMAT is osmium volume within the proximal epiphysis. (**k**) Distal BMAT is osmium volume from tibia-fibula junction to distal epiphysis. (**l**) Osmium content within the whole tibia bone marrow. Mean ± S.D. Two-tailed t-test, *P ≤ 0.05. Sample sizes: Male Cre- = 5–6, Male Cre +  = 4, Female Cre- = 7, Female Cre +  = 7. All ages were 4-weeks old.

*Ucp1*-Cre expressing mice were bred with mice carrying a floxed diphtheria toxin subunit A (DTA) gene, creating progeny (*Ucp1*-Cre, ROSA26^DTA/+^, referred to as *Ucp1*^Cre+/DTA+^) in which expression of UCP1 would drive intracellular expression of DTA, resulting in cell death. Confirming the efficacy of this model, at 4-weeks-old, *Ucp1*-Cre mediated expression of DTA led to complete ablation of BAT in the majority of the *Ucp1*^Cre+/DTA+^ mice (7/11 animals, Fig. 3a). Residual BAT in the remaining *Ucp1*^Cre+/DTA+^ mice was ~10% of the WT average. Inguinal and gonadal fat pad masses were equivalent between male *Ucp1*^Cre+/DTA+^ and control mice (Fig. 3b,c), however, female *Ucp1*^Cre+/DTA+^ mice displayed reduced iWAT and gWAT masses relative to their control littermates (−33% and −46%, respectively). Total body weight and tibia length were normal in male *Ucp1*^Cre+/DTA+^ mice, but reduced in female *Ucp1*^Cre+/DTA+^ mice, reflecting an overall reduction in body size in female but not male *Ucp1*^Cre+/DTA+^ animals (Fig. 3d,e). Confirming a *Ucp1*-Cre-mediated and DTA-induced cell death, a reduction of BAT, iWAT and gWAT tissues compared to the *Ucp1*^Cre-/DTA+^ controls remained after normalization of tissue weights to body mass (Fig. 3f–h). To quantify BMAT in *Ucp1*^Cre+/DTA+^ mice, decalcified tibias were stained with osmium tetroxide and imaged via microcomputed tomography (µCT, Fig. 3i). Regional analysis of BMAT volume was then performed and included the proximal epiphysis (rBMAT, Fig. 3j), distal tibia (cBMAT, Fig. 3k), and the total medullary cavity (Fig. 3l). Neither regional or total BMAT volume were reduced in *Ucp1*^Cre+/DTA+^ male or female mice. Instead, there was a slight trend toward increased BMAT volume in the distal BMAT and total BMAT measurements (Fig. 3k,l).

### Adipocytes within the developing skeleton express adiponectin, but not UCP1, by lineage tracing

**Figure 4:**
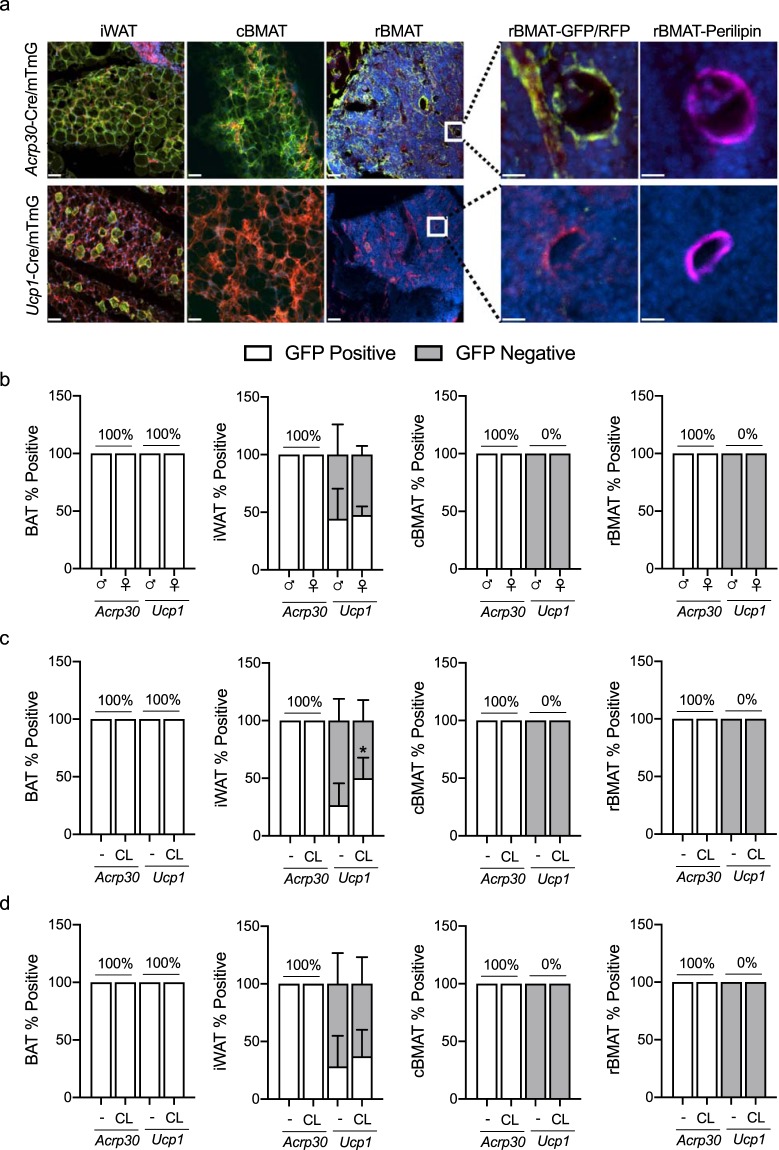
Adipocytes within the developing skeleton express adiponectin, but not UCP1, by lineage tracing. (**a**) Representative images of *Adipoq*^Cre+/mTmG+^ and UCP1^Cre+/mTmG+^ inguinal white adipose tissue (iWAT), constitutive bone marrow adipose tissue (cBMAT, caudal vertebrae), and regulated bone marrow adipose tissue (rBMAT, tibia). The presence of green (GFP-positive) cells indicates expression of Cre and conversion of the mTmG reporter. White boxes in images show inset for images to the right (GFP/RFP). Far right images (perilipin) demonstrate that the adipocytes in the GFP/RFP images are perilipin-positive. Images were acquired at 10X, scale bar is 50 µm or 25 µm for insets. (**b**–**d**) Quantification of *Adipoq*-Cre or *Ucp1*-Cre-mediated GFP expression in adipocytes relative to total number of adipocytes. White bars are the percent of adipocytes expressing GFP. Grey bars are the percent of adipocytes that do not express GFP (instead express RFP). Tissues collected from 3-week-old male and female mice (**b**) 16-week-old males (**C**) or females (**d**) treated with saline (−) or 0.3 mg/kg CL316,243 (CL) for 6-days. (**b**–**d**) Mean ± S.D. Sample sizes: (**b**) n = 5–6 (**c**) n = 3–5, (**d**) n = 3–7.

Expression of UCP1 in BMAT was then assessed using lineage tracing. *Ucp1*-Cre mice were crossed with mT/mG reporter mice to generate *Ucp1*^Cre+/mTmG+^ mice. In the presence of Cre, recombination leads to excision of tomato (RFP) and expression of GFP^25^. Thus, any cell having expressed Cre will change plasma membrane color from red to green. Adiponectin-Cre (*Adipoq*-Cre) mice were bred separately to mT/mG reporter mice (referred to as *Adipoq*^Cre+/mTmG+^) to serve as a positive control for this experiment. Confocal imaging of RFP and GFP in BAT, iWAT, cBMAT (tail) and rBMAT was completed in male and female mice aged 3-weeks and 16-weeks (Fig. 4). Confirming the efficacy of these models, 100% of the BAT adipocytes and approximately 45% of iWAT adipocytes expressed GFP in *Ucp1*^Cre+/mTmG+^ mice (age 3-weeks, Fig. 4b). In the *Adipoq*^Cre+/mTmG+^ mice, 100% of the BAT and iWAT adipocytes were GFP positive (Fig. 4b). GFP positive, *Ucp1*-Cre expressing cells could not be found among either cBMAT or rBMAT adipocytes (perilipin positive) of male or female mice (Fig. 4b). In contrast, all BMAT adipocytes, regardless of location, were GFP positive in the *Adipoq*^Cre+/mTmG+^ mice.

### ß3-adrenergic agonist CL316,243 does not induce UCP1 expression in adult BMAT

Treatment with ß3-adrenergic agonist CL316,243 is an established mechanism for inducing adipocyte beiging and UCP1 expression in rodents^26^. To determine whether BMAT adipocytes express UCP1 after CL316,243 treatment *in vivo*, *Ucp1*-Cre-mediated GFP expression was evaluated in 16-week-old male and female *Ucp1*^Cre+/mTmG+^ reporter mice, following six days of 0.3 mg/kg CL316,243 treatment (Fig. 4c,d). *Adipoq*^Cre+/mTmG+^ mice were used again as a positive control for GFP, and iWAT from CL316,243 treated mice served as positive control for *Ucp1*-cre mediated GFP induction. *Adipoq*-Cre-mediated GFP was uniformly expressed in BAT, iWAT and BMAT adipocytes of male and female *Adipoq*^Cre+/mTmG+^ mice, irrespective of CL316,243 treatment (Fig. 4c,d). *Ucp1*-Cre-mediated GFP was expressed in 100% of BAT adipocytes in both male and female *Ucp1*^Cre+/mTmG+^ (Fig. 4c,d). Approximately 27% of iWAT adipocytes in male saline-treated *Ucp1*^Cre+/mTmG+^ mice were GFP positive. With CL316,243 treatment, approximately 50% of iWAT adipocytes in male *Ucp1*^Cre+/mTmG+^ mice had *Ucp1*-Cre-mediated GFP expression, an 85% increase. In female saline-treated *Ucp1*^Cre+/mTmG+^ mice, approximately 28% of the iWAT adipocytes were GFP positive, increasing to 37% after treatment with CL316,243. In the *Ucp1*^Cre+/mTmG+^ bone marrow, neither rBMAT or cBMAT adipocytes (perilipin positive) expressed *Ucp1*-Cre-mediated GFP, regardless of sex or CL316,243 treatment.

### UCP1 and adiponectin mTmG reporter conversion occurs in non-adipocytes

**Figure 5:**
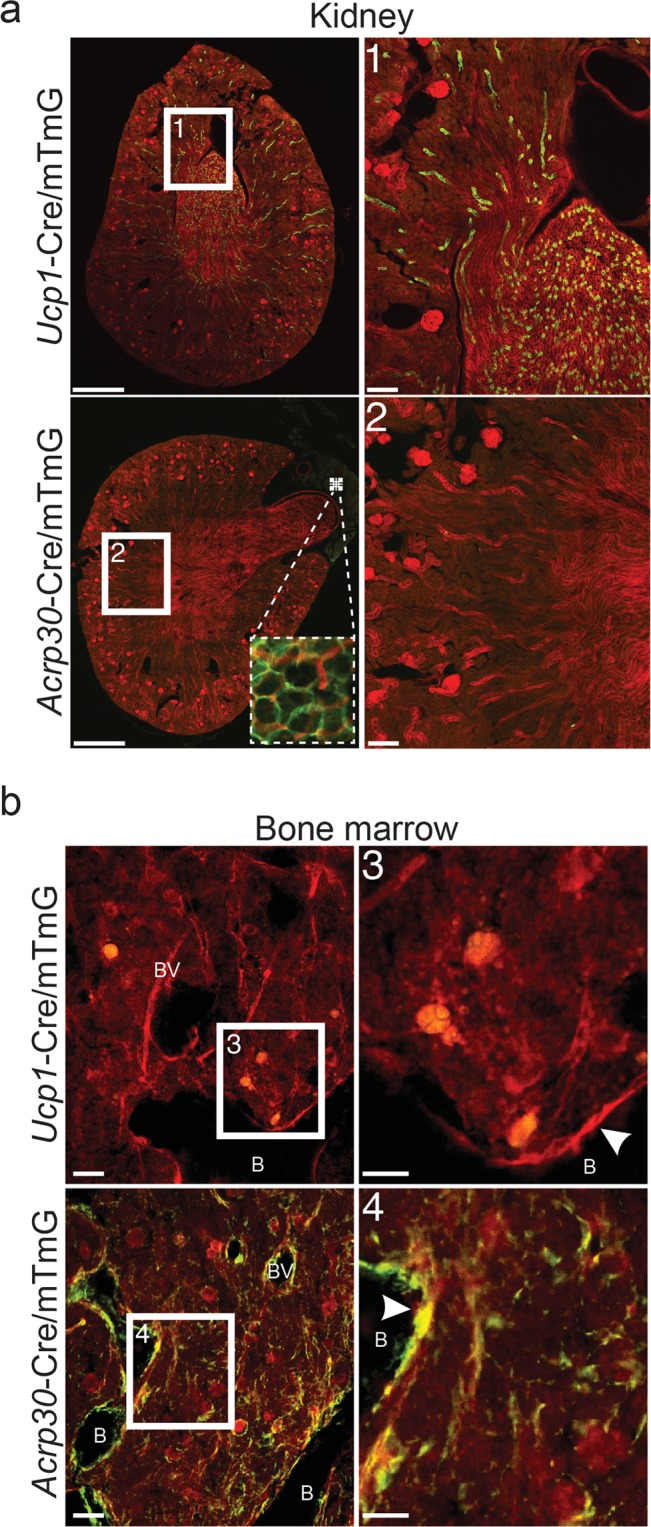
UCP1 and adiponectin mTmG reporter conversion occurs in non-adipocytes. (**a**) Representative images of kidneys from 16-week-old *Ucp1*^Cre+/mTmG+^ and *Adipoq*^Cre+/mTmG+^. The presence of green (GFP-positive) cells in *Ucp1*^Cre+/mTmG+^ images indicates expression of Cre and conversion of the mTmG reporter in kidney collecting ducts (non-adipocytes) of *Ucp1*^Cre+/mTmG+^ mice. Numbered white boxes show inset for numbered images to the right. Dashed boxes in the bottom left (*Adipoq*^Cre+/mTmG+^) image show targeted expression of *Adipoq*-Cre in peri-renal adipocytes. Images were acquired at 10x then stitched together, scale bars are 1 mm and 200 µm. (**b**) Representative images of bone marrow from 16-week-old *Ucp1*^Cre+/mTmG+^ and *Adipoq*^Cre+/mTmG+^ mice. The presence of green (GFP-positive) cells in *Adipoq*^Cre+/mTmG+^ images indicates expression of Cre and conversion of the mTmG reporter in bone lining cells (white arrowheads), cells along blood vessels, and cells along bone marrow reticular fibers of *Adipoq*^Cre+/mTmG+^ mice. Numbered white boxes show inset for numbered images to the right. “B” indicates location of bone. “BV” indicates location of blood vessels. Images were acquired at 10X, scale bars are 50 µm and 25 µm.

To verify the specificity of the *Ucp1*^Cre+/mTmG+^ and *Adipoq*^Cre+/mTmG+^ reporter mice, we also examined a subset of peripheral tissues. Liver, pancreas, spleen, lung, and intestine were negative for GFP expression in *Ucp1*^Cre+/mTmG+^ mice at 3- and 16-weeks. However, there was notable *Ucp1*-Cre-mediated GFP expression in cells of the kidney collecting ducts. GFP expression in the kidneys of *Ucp1*^Cre+/mTmG+^ mice was present regardless of age, sex or CL316,243 treatment (Fig. 5a, male and female, 3- and 16-weeks-old, with and without CL316,243, n = 34 total). By contrast, kidney, liver, pancreas, spleen, lung, and intestine were negative for *Adipoq*-Cre-mediated GFP expression in all conditions (*Adipoq*^Cre+/mTmG+^ male, female, 3-week, 16-week, +/− CL316,243, n = 30 total). However, widespread *Adipoq*-Cre-mediated GFP expression was observed in the stromal reticular cell network of the bone marrow, cells lining blood vessels within bone, and bone-lining cells of the femur and tibia in all *Adipoq*^Cre+/mTmG+^ mice regardless of age or treatment (Fig. 5b). As a control, neither the stromal reticular cell network of the bone marrow, vessel lining cells or bone lining cells expressed GFP in the *Ucp1*^Cre+/mTmG+^ mice. Gonadal tissue was also assessed at 16-weeks of age and was negative regardless of sex or CL316,243 treatment (n = 19, *Ucp1*^Cre+/mTmG+^; n = 18, *Adipoq*^Cre+/mTmG+^).

### Gene expression profiles in BMAT suggest enhanced roles in bone homeostasis and altered metabolism relative to WAT

**Figure 6:**
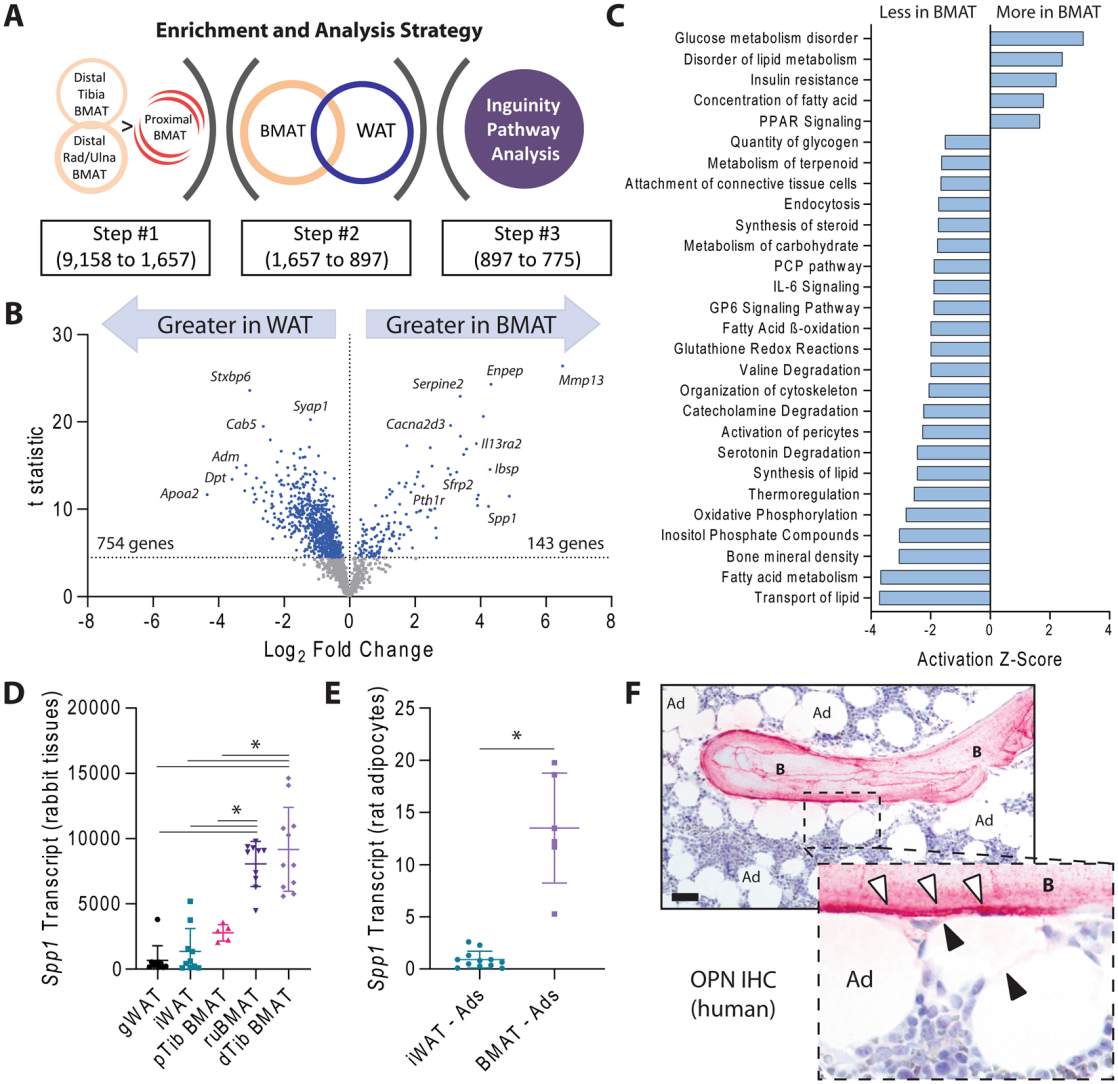
BMAT gene enrichment analysis. (**a**) Gene enrichment and analysis strategy. Of 9,158 detected transcripts, 1,657 were significantly increased in regions of high BMAT (distal tibia and radius/ulna) relative to low BMAT (proximal tibia). (**b**) Of these, 897 were differentially expressed relative to WAT (blue dots, full dataset in Supp Table 1). (**c**) Rabbit gene IDs of these differentially expressed transcripts were converted to human identifiers and used for Inguinity Pathway Analysis (full list available in Supp Table 2). (**d**) Linearized values for osteopontin (*Spp1*) transcript expression from rabbit whole tissues. 1-way-ANOVA with Tukey’s multiple comparisons test, *p < 0.05. (**e**) *Spp1* transcript from purified inguinal white adipose tissue (iWAT) and tail bone marrow adipocytes (BMAT) normalized to the geometric mean of reference genes *Tbp* and *Ppia*. Two-tailed t-test. *p < 0.05. (**f**) Representative osteopontin immunohistochemistry of human bone and bone marrow. B = bone, Ad = bone marrow adipocytes, white arrows = osteopontin immunostaining in bone (pink), black arrows = osteopontin immunostaining in bone marrow (pink). Scale bar is 50 µm.

To identify novel and emerging roles for BMAT adipocytes in both skeletal and whole-body homeostasis, we performed microarray analysis on white adipose tissues (inguinal and gonadal) and bone marrow adipose tissues from the proximal tibia, distal tibia, and radius/ulna of adult rabbits^27,28^. Unlike rodents, both rabbit and human bones have haversian canals, making this model of potentially increased relevance to the human condition^29^. Like humans, rabbit bone marrow also develops extensive intramedullary BMAT which is concentrated around the central vascular cannals within the long bones^27^. Using a serial gene enrichment strategy, we identified 1,657 transcripts out of 9,158 that were enriched in regions of high BMAT (distal tibia and radius/ulna) relative to low BMAT (proximal tibia) (Fig. 6a,b). This included genes such as Wnt modulator *Sfrp2*, cell adhesion ligands such as *Ibsp*, and PTH receptor *Pth1r*, but not *Ucp1* (Fig. 6b). Of these, 897 genes were differentially expressed relative to WAT and, of those, 775 were able to be mapped to corresponding human IDs for Inguinity pathway analysis (Supplementary Table 1). This revealed genetic evidence for differences in BMAT metabolism including decreased glycerol content, insulin resistance, decreased fatty acid metabolism, and decreased thermoregulation (Fig. 6c, full list in Supplementary Table 2). Genes related to lipid synthesis and transport pathways were also reduced in BMAT. Cellular pathway analysis suggested that this may be due to underlying reductions in fatty acid β-oxidation and oxidative phosphorylation (Fig. 6c). Degradation of biomolecules including valine, catecholamines, and serotonin was also predicted to be reduced (Fig. 6c). Lastly, genes involved in skeletal pathways related to decreased bone mineral density were enriched in BMAT. Of these, the osteopontin gene *Spp1* was among the most differentially expressed relative to WAT and had among the highest transcript levels detected (Fig. 6d). Thus, to validate our array and determine whether *Spp1* was differentially expressed in the BMAT adipocyte itself, we performed qPCR analysis of purified BMAT and iWAT adipocytes from rats. Comparable to rabbit whole tissues, we found that expression of *Spp1* was 15-fold higher in purified BMAT adipocytes relative to iWAT (Fig. 6e). Lastly, we visualized osteopontin expression at the protein level in human skeletal biopsies. Consistent with previous reports^30^, we observed robust staining for osteopontin within the bone matrix along the cement lines and around the osteocytes (Fig. 6f). When present, bone lining cells were also noted to be positive. In many regions, BMAT adipocytes were situated immediately adjacent to osteopontin-rich bone surfaces and, in some cases, demonstrated faint cytoplasmic reactivity (Fig. 6f).

## Discussion

In healthy individuals, BMAT can occupy up to 70% of the medullary cavity and accounts for ~8% of total body fat^1,6^. Despite the abundance of BMAT, its function remains unclear. The concept of BMAT functioning as an energy depot, similar to WAT adipocytes, seems relevant in areas of high hematopoietic activity and bone turnover, such as the proximal tibia and distal femur (rBMAT region), and is supported by recent publications. Electron microscopy (EM) studies have shown BMAT adipocytes to interact with myeloid/granulocyte cells and erythroblast islands^11^. The same EM study showed extensions of lipid droplet-filled cytoplasm from BMAT adipocytes adjacent to active osteoblasts^11^. By contrast, regions of confluent constitutive BMAT adipocytes are selectively retained and often resist conventional lipid mobilization cues (reviewed in^31^). Our microarray study provides a novel list of 897 candidate genes that may underlie these functional relationships and differences in metabolic responsiveness (Fig. 6, Supplementary Table 1). Beyond this, in pathologic conditions, BMAT adipocytes have been shown to facilitate and fuel bone tumor progression (reviewed in^32^). Less clear is whether BMAT adipocytes also have ‘beige’-like properties, more specifically, whether these adipocytes express UCP1 and therefore have the capacity for thermogenesis. Several attempts have been made to determine whether BMAT adipocytes do indeed express UCP1, but collectively have yielded conflicting results. To overcome this, we sought to track UCP1 expression in BMAT at the single cell level using multiple rodent models.

Deciphering whether BMAT adipocytes express UCP1 is of importance because it informs the function of these cells. The capacity of BMAT adipocytes to undergo UCP1-mediated adaptive thermogenesis would have multiple advantages. First, positioning of thermogenic cells within stem cell niches may protect hematopoietic cells and therefore blood cell production from cold exposure in distal appendages. Second, the shunting of energy substrates toward adaptive thermogenesis has the benefit of lowering glucose and lipid load. Thus, clearance of lipids via adaptive thermogenesis may also protect hematopoietic and bone remodeling niches from lipotoxicity. As previously discussed, several lines of evidence both support and refute the existence of UCP1 expression in BMAT adipocytes. Briefly, *Ucp1* transcript has been detected in whole bone RNA preparations^14–17^, however, this type of tissue preparation is susceptible to contamination by adipocytes near the periosteal surface which we now show have the potential to express UCP1 (Fig. 2). Further, comparison of *Ucp1* transcript amount in these bone preparations is >10,000 fold less when compared to BAT^14,16^. It is also unknown whether *Ucp1* transcript would result in UCP1 protein expression. One report did show putative UCP1 protein expression by IHC in comparable appearing, ‘multilocular’ BMAT adipocytes within the lumbar vertebrae of 3-week old mice^18^. However, in this report, we show that immunostaining of bone marrow can easily provide false positive results, particularly in the absence of UCP1-KO tissue controls (Fig. 2). There are also reports that UCP1 protein is not expressed in BMAT, even after beiging induction by methionine-restriction^20,23^.

In this study, three approaches were taken to specifically address whether BMAT adipocytes have the potential for UCP1 expression: 1) immunohistochemical detection of UCP1 protein in intact tissue, 2) *Ucp1*-Cre-driven and DTA-mediated cell ablation, and 3) *Ucp1*-Cre-mediated lineage tracing. Immunohistochemistry for UCP1 expression provided inconclusive results. Specifically, while cBMAT adipocytes were clearly negative, non-specific DAB precipitation was present in hematopoietic-rich bone marrow of both control and UCP1-knockout mice making UCP1 expression in rBMAT cells difficult to assess. However, *Ucp1*-Cre driven cell ablation and lineage tracing provided compelling data that BMAT adipocytes, regardless of subregion, do not express UCP1. *Ucp1*-Cre driven expression of DTA resulted in loss of brown adipocytes and a subpopulation of iWAT adipocytes, but there was no detectable loss of BMAT adipocytes as assessed by 3-dimensional, regional analysis of BMAT in the proximal tibia, distal tibia and whole tibia. While these experiments cannot rule out the possibility of a loss of a Ucp-1 positive population that is replaced by a Ucp-1 negative BMAT population this possibility is not supported by our lineage tracing experiments. Lineage tracing using *Ucp1*-Cre and the mTmG reporter found that *Ucp1*-Cre successfully induced GFP expression in BAT and subpopulations of iWAT but did not induce GFP expression in BMAT of the femur, tibia or tail vertebrae regardless of age, sex, or induction by beiging factor CL316,243. A limitation of reporter models is that they rely on induction of the *Ucp1*-Cre. Cre recombinase models can have off-target expression or insufficient recombination. However, it is unlikely that these concerns would change the outcome of our study. First, there was no detectable difference in BMAT volume in the *Ucp1*^Cre+/DTA+^ model nor GFP-positive BMAT in *Ucp1*^Cre+/mTmG+^ mice, ruling out mis-expression in BMAT. Second, sufficient *Ucp1*-Cre-mediated recombination is supported by loss of peripheral brown and beige adipocytes in *Ucp1*^Cre+/DTA+^ mice, and conversion of the mTmG reporter in these adipocytes, as well as subpopulations of adipocytes near the periosteal surface in *Ucp1*^Cre+/mTmG+^ mice. Further, conversion of the mTmG reporter by the *Adipoq*-Cre served as a positive control for the ability of BMAT adipocytes to support Cre-mediated recombination.

Lack of UCP1 expression despite a multilocular morphology could indicate that BMAT adipocytes transition from an early multilocular stage to a mature unilocular morphology; a physiologic transition similar to the fusion of immature lipid droplets in white adipocytes (reviewed in^24^). In this report, we show that the frequency of multilocular BMAT adipocytes is comparable across anatomical sites and is preserved between ages 3-weeks to 9-weeks old (~5%). Also, that this frequency is comparable to that reported for 12-week-old C3H/HeJ mice^7^. Together, these findings indicate that ‘multilocularity’ is stable across age and mouse strain. If adipocytes transition through a multilocular state, then it can be extrapolated that BMAT adipocyte turnover occurs at constant rate despite location or age. In addition, multilocularity could imply that these cells are undergoing simulated lipid droplet remodeling (lipolysis) and therefore have the ability to function as a local fuel reservoir. In support of this hypothesis, we recently demonstrated, using electron microscopy, that BMAT adipocytes directly interact with cells of the myeloid/granulocyte lineage, erythroblast islands, and active osteoblasts^11^. Additionally, there are published reports showing BMAT provides fuel to cancer cells (reviewed in^32^) and that inflammatory arthritis is associated with BMAT depletion^33^. As demonstrated by our microarray analysis and previous reports^7,28,34^, the regulation of BMAT energy storage and partitioning is unique when compared to peripheral WAT. For example, pathway analysis indicated that BMAT likely has reduced quantity of glycogen relative to WAT, a finding that has previously been demonstrated by electron microscopy during BMAT adipogenesis^34^. BMAT was also noted to be insulin resistant, which has recently been confirmed in both rodents and humans^28^.

Lastly, lineage tracing studies have yielded mixed results regarding identification of a ‘beige’-like BMAT adipocyte. Specifically, BMAT adipocytes have been shown to express *PdgfRα*, a mediator of adipocyte progenitor commitment to a beige adipocyte^19^, following irradiation or rosiglitazone treatment (discussed in^20^). However, myogenic factor-5 (*Myf5*), which is expressed in the majority of brown adipocyte progenitors and a minority of beige progenitors^21,22^, was not detected in BMAT progenitors (discussed in^20^). In our study, lineage tracing clearly showed that BMAT adipocytes express the adipocyte marker adiponectin, but do not express the traditional beige cell marker *Ucp1*. However, *Ucp1*-Cre mediated conversion of the mTmG reporter did show *Ucp1*-Cre expression in cells of the kidney collecting ducts. Assessment of UCP1 expression in the mouse kidney has been evaluated by other labs. There is one report supporting UCP1 expression in mouse kidney^35^, however, there are multiple reports indicating mammalian kidneys do not express UCP1^36–38^. Thus, this is a point that warrants further clarification. Similarly, though *Adipoq*-Cre is not expressed in the stromal vascular fraction of adipose tissue^39^ or skin stromal cells^40^, *Adipoq*^Cre+/mTmG+^ mice demonstrated prevalent *Adipoq*-Cre expression in bone lining cells, perivascular cells within bone, and cells within the reticular stromal network, a finding consistent with other reports^41,42^. This continuity of expression between the stromal-reticular network and BMAT is also emphasized in our microarray data. Specifically, we chose to examine osteopontin (*Spp1*) since it was one of the most highly expressed transcripts in our enrichment analysis (#5/897) and was also among the most differentially expressed relative to WAT (~90-fold higher). We also found evidence of differential *Spp1* expression in purified rat BMAT adipocytes relative to iWAT. Osteopontin (*Spp1)* is broadly expressed across tissues^43^, including bone marrow stromal and lining cells^30,44^ and adipocytes^43^. There is evidence for both secreted and cell-autonomous functions with downstream impacts on mineralization, lipid metabolism, and metastasis^43,45–47^. Future work needed to clarify the role of *Spp1* and other candidate genes at the BMAT cell level across species and to characterize their contribution to BMAT function.

## Conclusion

Our data demonstrate that BMAT does not express UCP1 during development, in maturity, or after β3-agonist treatment. This provides important information about the origin and potential functions of BMAT. Though our data largely rules out the potential for UCP1-mediated thermogenesis in BMAT adipocytes, UCP1-independent mechanisms for adaptive thermogenesis have recently been described in both adipose tissue and muscle and warrant additional investigation. Specifically, lipid and creatine cycling are mechanisms allowing UCP1-independent thermogenesis in fat^48^, while calcium cycling has been shown to support alternative thermogenesis in both fat and muscle^49–52^. It should also be considered that BMAT represents a unique subtype of adipose tissue with endocrine and paracrine functions distinct from both brown and white adipose tissues. Clarification of the genes and proteins expressed by BMAT adipocytes will advance our understanding of BMAT function and its regulation in both health and disease.

## Materials and Methods

### Animal models and use

Institutional guidelines for the handling and experimentation with animals were followed for this study. Mouse studies were approved by the animal use and care committee at Washington University (Saint Louis, MO, USA). To generate *Ucp1*-Cre, ROSA26^DTA/+^ (*Ucp1*^Cre+/DTA+^) animals, heterozygous *Ucp1*-Cre+ males (Jackson Labs #024670) were bred to female diphtheria toxin subunit A (ROSA26^DTA/DTA^) homozygous mice (Jackson Labs #009669). Due to loss of brown adipose tissue and impaired thermoregulation, *Ucp1*^Cre+/DTA+^ animals do not survive when born at room temperature and thus were bred and housed at thermoneutrality (30 °C). *Ucp1*^Cre+/DTA+^ animals have a head tilt phenotype. Given there is no change in BMAT volume in *Ucp1*^Cre+/DTA+^ animals, this finding does not affect the conclusions of this paper. To generate *Ucp1*-Cre, ROSA26^mTmG/+^ and *Adipoq*-Cre, ROSA26^mTmG/+^ (*Ucp1*^Cre+/mTmG+^ and *Adipoq*^Cre+/mTmG+^) animals, heterozygous *Ucp1*-Cre+ or *Adipoq*-Cre+ males (Jackson Labs #024670 and #028020) were bred to homozygous mTmG (ROSA26^mTmG/mTmG^) female mice (Jackson Labs #007676). *Ucp1*^Cre+/mTmG+^ or *Adipoq*^Cre+/mTmG+^ reporter animals have no deficiencies in thermoregulation and were thus bed and housed under standard conditions at 22 °C. All transgenic mice were maintained on an a C57BL/6J background (Jackson Labs #000664). All animals were housed in a vivarium with a 12-hour light/dark cycle and fed standard chow *ad libitum* (PicoLab 5053, LabDiet).

Rabbit and rat studies were approved by the University of Michigan Committee on the Use and Care of Animals. Housing, monitoring, and euthanasia information for both the rabbits^27^ and rats^4^ included in this study has been previously reported. Briefly, male New Zealand White rabbits from Harlan Laboratories (Haslett, MI, USA) were euthanized at two ages, 13-weeks and 22-weeks, for tissue collection and analysis. Rabbit care was provided by the Unit for Latoratory Animal Medicine. Rabbits were housed at 22 °C on a 12-hour light/dark cycle and fed high-fiber diet (LabDiet: 5326) *ad libitum* (13-week old rabbits, body mass 2.70 ± 0.06 kg, n = 6) or a measured 100 g/day starting at 15-weeks of age (22-week old rabbits, body mass 3.17 ± 0.09 kg, n = 5). Sixteen-week-old male Sprague–Dawley rats were obtained from Charles River Laboratories (strain code: 400). Rats were were housed at 22 °C on a 12-hour light/dark cycle and fed standard chow.

### CL316,243 treatment regimen

Male and female *Ucp1*^Cre+/mTmG+^ and *Adipoq*^Cre+/mTmG+^ reporter animals aged 15-weeks received six 0.03 mg/kg CL316,243 (Sigma) or saline injections over the course of eight days. On the ninth day, animals were perfused with 10% buffered formalin and tissues collected. 12-week-old C3H/HeJ mice received 5 µg CL316,243 injections daily for 3 days. After 72-hours, mice were euthanized and tissues collected.

### Tissue collection (mouse)

Following euthanasia, brown adipose tissue (BAT), inguinal white adipose tissue (iWAT), gonadal white adipose tissue (gWAT) and tibias were collected from *Ucp1*^Cre+/DTA+^ animals. Tibia length was determined using a digital caliper. Tibias were post-fixed for 24-hours then decalcified in 14% EDTA, pH 7.4 for 3 weeks. Tissues from 3-week and 9-week-old C57BL/6J mice for paraffin embedding were post-fixed for 24-hours in 10% neutral buffered formalin (NBF) then stored in PBS. Prior to collection of *Ucp1*^Cre+/mTmG+^ and *Adipoq*^Cre+/mTmG+^ tissues, mice were perfused with 10% NBF. Harvested tissues were then post-fixed for an additional 24-hours in 10% NBF. Paraffin embedding was performed by the WUSM Musculoskeletal Histology and Morphometry Core. All paraffin sections were 5 µm thick. Prior to embedding, bones were decalcified in 14% EDTA, pH 7.4 for 2-weeks.

### Biopsy specimens (human)

Human control biopsies from the iliac crest were included from the pathological biobanks at Vejle Hospital in accordance with the approval by the Danish National Committee on Biomedical Research Ethics, journal no. S-20070121, as described previously^53^. The biopsies were diagnostic jamshidi biopsies obtained from patients formerly undergoing an examination for a hematological disorder, showing no skeletal disorders. The biopsies included in the study originated from patients not receiving any prior medications known to affect the bones and not declining use of their tissues for research in the National Tissue Application Register. The biopsies were blinded after inclusion (sex and age unknown).

### Paraffin immunohistochemistry

Prior to hematoxylin/eosin or immunohistochemistry, sections were deparaffinized in xylene and hydrated in an ethanol gradient and distilled water.

Perilipin and endomucin immunohistochemistry *(mouse)* was performed as described previously^11^. Briefly, antigen retrieval was performed in 10 mM sodium citrate buffer (pH 6.0, 20-minutes, 90-95 °C) prior to permeabilization in 0.2% Triton-X in PBS and block in 10% donkey serum in TNT buffer (0.1 M Tris-HCL pH 7.4, 0.15 M sodium chloride, 0.05% Tween-20). Sections were co-stained with antibodies against endomucin (BioLegend, 1:500) and perilipin (1:400, Progen GP29) in TNT buffer containing 2.5% donkey serum overnight at 4 °C. Secondary antibodies (1:200 donkey anti guinea pig Cy3 and 1:200 donkey anti rat Alexa 488; Jackson ImmunoResearch) in TNT buffer were applied for 1-hour at room temperature. All washes between steps were performed in TNT buffer. Nuclei were counterstained with 1 μg/mL DAPI (Sigma). Slides were imaged on an Olympus FV1200 confocal microscope.

For UCP1 immunohistochemistry *(mouse)*, tissue samples were permeabilized for 10-minutes and blocked for 1.5-hours with the buffer provided from Vectastain Elite ABC kit (Vector Laboratories). Primary antibody cocktail (1:2,000 UCP1, Abcam polyclonal ab10983) was made in TNT buffer containing 2% donkey serum and incubated overnight at 4 °C. Tissue sections were washed in TNT followed by quenching of endogenous peroxidases with 0.3% hydrogen peroxide in PBS for 30-minutes. Sections were washed 3x again in TNT prior to incubation with kit-provided secondary antibody (ImPRESS) for 30-minutes. After the secondary antibody, tissue samples were again washed 3x in TNT and 2x in PBS. Tissues were incubated with DAB substrate solution for 30-seconds for stain development, then washed in water. After a final wash, samples were stained with hematoxylin, dehydrated through a reverse ethanol gradient and mounted with Permount mounting media. Images were acquired using Nanozoomer 2.0-HT system.

For osteopontin (SPP1) immunohistochemistry *(human)*, antigen retrieval was performed by heating samples in Tris/EDTA (pH 9.0) for overnight at 60 °C. Tissue sections were blocked for 20 minutes using 1% casein in TNT buffer. Primary antibody (1:25 osteopontin, R&D Systems, BAF1433) was made in TNT buffer containing 1% casein and incubated 1-hour at room temperature. Secondary antibody (1:50 donkey anti Goat Alkaline Phosphatase, Jackson ImmunoResearch 705-056-147) was made in TNT buffer and incubated for 30 minutes at room temperature. Chromogen staining was made with Liquid Permanent Red (DAKO). All washes between steps were performed in TNT buffer. Nuclei were counterstained with Meyer hematoxylin. Slides were imaged using an upright DMRXAZ microscope (Leica) and a UC30 camera (Olympus).

Quantification of multilocular bone marrow adipocyte frequency was performed on H&E stained tibia and femur sections from 3-week and 9-week-old C57BL/6J mice. As previously described^7^, BMAT adipocytes were considered multilocular if they contained three or more smaller lipid droplets associated with a large lipid droplet. All adipocytes within the epiphysis (distal for femur, proximal for tibia), metaphysis (mid-diaphysis to growth plate for femur, growth plate to tibia-fibula junction for tibia), or distal tibia were counted. Two tissue sections per mouse were analyzed and the data averaged.

### Frozen immunohistochemistry

*Ucp1*^Cre+/mTmG+^ and *Adipoq*^Cre+/mTmG+^ reporter animals were anesthetized with ketamine/xylazine cocktail (80 mg/kg and 5 mg/kg, respectively) perfused with 10% NBF prior to euthanasia. Harvested tissues were post-fixed for 24-hours in 10% NBF then washed in PBS. Bones were decalcified using 14% EDTA, pH 7.4 for 14-days. Tissues were embedded in OCT mounting media and sectioned at 50μm (tibia, iWAT, kidney) or 100μm (tail) using a cryostat (Leica). Tissue sections were rinsed 3x in TNT and blocked for 1-hour with 10% donkey serum in TNT buffer (0.1 M Tris-HCL pH 7.4, 0.15 M sodium chloride, 0.05% Tween-20). Primary antibody cocktail (1:1,000 chicken anti-GFP; 1:500 rabbit anti-RFP; 1:1,000 guinea pig anti-perilipin; Abcam ab13970, ab62341 and Progen GP29, respectively) was made in TNT buffer and incubated overnight at 4 °C. Secondary antibody cocktail (1:500 donkey anti-guinea pig Alexa 647; 1:500 donkey anti-chicken Alexa 488; 1:500 donkey anti-rabbit Alexa 594; Jackson ImmunoResearch 706-605-148, 703-545-155, and 711-585-152 respectively) was made in TNT buffer and incubated for 24-hours at 4 °C. Images were acquired using a Nikon Spinning Disk confocal microscope. Quantification of GFP/RFP positive adipocytes was performed in iWAT, gWAT, BAT and three regions of BMAT including tail vertebrae, femur, and proximal tibia (Fig. 4 and data not shown). All perilipin positive adipocytes in a full length, 50–100 μm section were assessed for each animal.

### Osmium staining and micro-computed-tomography (µCT)

Tibias from 4-week-old *Ucp1*^Cre+/DTA+^ and littermate controls were decalcified in 14% EDTA, pH 7.4 for 3-weeks then stained with osmium tetroxide and imaged as described previously^54^. Briefly, decalcified bones were incubated in a PBS solution containing 1% osmium tetroxide (Electron Microscopy Sciences) and 2.5% potassium dichromate (Sigma) for 48-hours. Following thorough washing, osmium-stained bones were embedded in 2% agarose and scanned a 10 µm voxel resolution using a Scanco μCT 40 (Scanco Medical AG). Analysis and 3-D rendering of the μCT data sets were performed using Scanco software, and a threshold of 400. Analysis regions included the tibia proximal epiphysis, distal tibia (tibia-fibula junction to distal epiphysis) and total medullary cavity.

### Microarray enrichment analysis (rabbit)

RNA was isolated from whole white adipose tissues (inguinal, iWAT; gonadal, gWAT) and bone marrow adipose tissues (distal tibia, dBMAT; radius/ulna, ruBMAT; proximal tibia, pBMAT) of rabbits as described previously^27^. Briefly, rabbit tissues were frozen on dry ice, pulverized in liquid nitrogen with a mortar and pestle, and lysed in Stat60 reagent (Tel-Test Inc) by manual disruption with a needle and syringe. For microarray, purified rabbit RNA was digested on-column with DNase I and cleaned using the Qiagen RNeasy kit (Qiagen) as recommended by the manufacturer. Total RNA was then submitted to the microarray core at the University of Michigan. The samples were screened for quality and processed in the microarray facility using custom rabbit Affymetrix arrays and the IVT Express kit (Affymetrix). Samples that did not meet minimum RNA quality or concentration standards were excluded from microarray analysis. As a QC measure, the distribution of probe intensities and the 5’ to 3’ degradation profiles were checked to be consistent across samples. The core’s statistician used RMA, from the Affy package of bioconductor, to fit log_2_ expression values to the data^55^. Weighted, paired, linear models were then fit and contrast computed using the limma package^56^. Weighting was done using a gene-by-gene algorithm designed to down-weight chips that were deemed less reproducible^57^. Probe-sets with a variance over all samples less than 0.05 were filtered out, leaving 9,158 transcripts for analysis across both cohorts (Fig. 6a). The data discussed in this publication have been deposited in NCBI’s Gene Expression Omnibus^58^ and are accessible through GEO Series accession number GSE138690. To enrich for BMAT adipocyte-associated transcripts, gene expression in regions of high BMAT content (distal tibia BMAT and radius/ulna BMAT) were compared to a region of relatively decreased BMAT (proximal tibia BMAT). P-values were adjusted for multiple comparisons using the Holm-Sidak method with alpha set at 0.05, resulting in identification of 1,657 transcripts that were statistically enriched in regions of high BMAT. From there, expression of these transcripts in BMAT (all dBMAT and ruBMAT) was compared to all white adipose tissues (all gWAT and iWAT) to generate a list of genes with high probability of differential expression between BMAT and WAT. As before, P-values were adjusted using the Holm-Sidak method and 897 transcripts were retained for further pathway analysis (Fig. 6a, Supplemental Table 1). To ensure maximum compatibility with the Inguinity Pathway Analysis software, rabbit gene identifiers were converted to their corresponding human homologues using the ‘BetterBunny’ algorithm^59^. Homology-based human IDs were identified for 775 out of 897 genes which were then used for pathway analysis (Fig. 6a, Supplemental Table 1).

### Adipocyte purification and qPCR analysis (rat)

Cell isolation, RNA purification, and qPCR analysis was completed as previously described^4^. Briefly, twelve 16-week-old male Sprague–Dawley rats were used to purify and isolate 6 BMAT adipocyte samples from caudal vertebrae (two animals pooled per sample) and 12 floated adipocyte preparations from inguinal WAT (one animal per sample) using a modified collagenase digestion protocol^4^. Floated adipocytes were collected and lysed using Stat60 reagent (Amsbio, Cambridge, MA, USA) to isolate total RNA. RNA was reverse-transcribed to cDNA (Applied Biosystems, Carlsbad, CA) and quantitative PCR was performed using qPCRBIO SyGreen mix, Hi-Rox, on an Applied Biosystems real-time PCR detection system. Gene expression was calculated based on a cDNA standard curve within each plate and normalized to the expression of the geometric mean of TATA-binding protein (*Tbp*) and cyclophilin A (*Ppia*) messenger RNA.

### Statistics

Statistical analysis was performed using GraphPad Prism (GraphPad Software). Statistical tests are detailed in the corresponding figure legend. A p-value of <0.05 was considered statistically significant.

## Supplementary information

Supplementary information

Supplementary information

Supplementary information

## Data Availability

All data contained within this manuscript are available upon request.
