# Supplementary material for: Bone marrow adipose tissue does not express UCP1 during development or adrenergic-induced remodeling
Source: Sci Rep. 2019 Nov 22;9:17427. doi: 10.1038/s41598-019-54036-x (PMC6874537; doi:10.1038/s41598-019-54036-x)
Supplement: Supplementary file 1 — Supplementary information [file 41598_2019_54036_MOESM1_ESM.docx]

Supplementary Table 1. Differentially expressed transcripts in BMAT vs WAT.

Supplementary Table 2. Inguinity Pathway Analysis results.
